# Selective Anti-melanoma Effect of Phosphothioated Aptamer Encapsulated by Neutral Cytidinyl/Cationic Lipids

**DOI:** 10.3389/fcell.2021.660233

**Published:** 2021-06-28

**Authors:** Jing Wu, Shuhe Wang, Xiang Li, Qi Zhang, Jie Yang, Yuan Ma, Zhu Guan, Zhenjun Yang

**Affiliations:** State Key Laboratory of Natural and Biomimetic Drugs, School of Pharmaceutical Sciences, Peking University, Beijing, China

**Keywords:** aptamer, phosphorothioate, cytidinyl lipid, hnRNP A2/B1, Liposome

## Abstract

BC15-31 is a DNA aptamer that targets heterogeneous nuclear ribonucleoprotein A1 (hnRNP A1), which plays a crucial role in the process of pre-RNA maturation and is also essential for the rapid proliferation of tumor cells. In this research, we modified BC15-31 with a phosphorothioate (PS) backbone, LNA, and 2-*O*-MOE to enhance its stability and target affinity. In addition, a neutral cytidinyl lipid (DNCA) and a cationic lipid (CLD) were mixed to encapsulate modified aptamers with the aim of improving their cell permeability with low toxicity. Under the DNCA/CLD package, aptamers are mainly distributed in the nucleus. A modified sequence WW-24 showed an excellent selective anti-melanoma (A375 cells, ∼25 nM, 80%) activity, targeted to both hnRNP A1 and hnRNP A2/B1 found by the BLI experiment, and induced more early and late apoptosis *in vitro*, which also showed stronger antitumor effect and longer accumulation time *in vivo*. These results provide a new strategy for further clinical applications.

## Introduction

Aptamers, which Szostak and Ellington first reported in 1990, are single-stranded oligonucleotides or peptides. The oligonucleotide aptamer can fold into a stable 3D structure through intramolecular base interactions in order to specifically bind to its target, which could be a metal ion, a small molecular compound, a nucleic acid, or a protein (Mascini et al., 2012); this binding occurs through π-stacking, H-binding, and/or electrostatic interaction (Hermann and Patel, 2000). The classic structures of oligonucleotide aptamers predominantly include stem loops (Tok et al., 2000), internal loops, pseudoknots (Park et al., 2008), kissing complexes (Chovelon et al., 2018), hairpin structures (Wen et al., 2016), and G-quadruplex structures (Yang et al., 2017; Zhou et al., 2018). Compared with antibodies, aptamers have multiple advantages: (1) the screening process of aptamers can be performed faster and easier *in vitro*, (2) the synthesis of aptamers is easy to control and not easily contaminated by bacteria and viruses, (3) aptamers have low impacts on immunogenicity, (4) the thermal stability of aptamers is better than that of antibodies (for example, a DNA aptamer can be stored for a long time at room temperature), and (5) aptamers are easy to be modified and can be covalently conjugated with fluorescent groups or other multifunctional groups for disease diagnosis or biochemical detection. Although aptamers have many advantages, they are rarely used as drugs for systemic administration because of their high electronegativity, hydrophilicity, and high molecular weight as oligonucleotides, making it challenging to penetrate cell membranes (Hoerter and Walter, 2007). Moreover, the half-life of naked nucleic acid in serum is about 15 min due to the degradation of the nuclease that is widely present in the blood, skin, and other organs (Sahay et al., 2010). The only aptamer-based drug approved by the FDA occurred in 2004, which was for the treatment of neovascular (wet) age-related macular degeneration (AMD). Using a local injection of high doses of pegaptanib sodium overcomes the problems above. The cationic liposomes are commonly used to protect the oligonucleotides *in vivo* and to improve their trans-membrane ability to achieve systemic administration. Nevertheless, the toxicity of cationic liposomes as nucleic acid drug carriers *in vivo* remains one of the obstacles (Filion and Phillips, 1997; Knudsen et al., 2015; Cui et al., 2018).

Heterogeneous nuclear ribonucleoproteins A1 (hnRNP A1) and A2/B1 (hnRNP A2/B1) are the most important members of the family. Both of them are often found overexpressed in many tumor cells (Pino et al., 2003; Ushigome et al., 2005). Because of its RNA-binding motifs (Xu et al., 1997; Ding et al., 1999), hnRNP A1 is also able to bind to G-quadruplexes or G-rich telomeric DNA (Xodo et al., 2008; Neriec and Percipalle, 2018) and plays an important role in stimulating telomerase activity (Zhang et al., 2006) and the maturation of pre-mRNA (Burd and Dreyfuss, 1994; He et al., 2005). The hnRNP A2/B1 is related to the occurrence and development of apoptosis inhibition and the invasion of tumor cells. Downregulation of hnRNP A1 or hnRNP A2/B1 by siRNA can decrease the invasion and migration of tumor cells, thus hinting that they are important for some tumor-related processes (Li et al., 2012; Dowling et al., 2015). hnRNP A2/B1 is also crucial for gene expression and mRNA stability and participates in controlling the protein translation (Zhang et al., 2017).

BC15 (74 nt) is a DNA aptamer that was found by tissue slide-based SELEX, which has a high affinity to hnRNP A1 and A2/B1 (Li et al., 2009), showing better antiproliferation activity in the HepG2 cell line (Ku et al., 2015). We found that the core sequence of BC15, BC15-31 (31 nt), showed higher target affinity and further enhanced bioactivity and stability with isothymidine (isoT) modification at 5′- and 3′-terminal regions (Li et al., 2018). Application of neutral cytidinyl lipid DNCA, through H-binding and π-π interaction, to reduce or avoid the use of cystine-based cationic lipid CLD (Ma et al., 2016), gave a safe and effective delivery system for oligonucleotides (Ma et al., 2018, 2019). In this study, phosphorathiolation (PS), LNA (ribose locked by a *O*-2′-C4′-methylene linkage), and 2′-methoxyethoxy (2′-*O*-MOE) modification strategies were used to improve the stability and target affinity of the aptamer BC15-31. With the mixture of DNCA and CLD as a carrier, a modified aptamer WW-24 (25 nM) showed higher target affinity and serum stability, which entered the nucleus, exerting a selective anti-melanoma activity, which could target hnRNP A1 and A2/B1. It also showed better antitumor effect and longer accumulation time *in vivo*.

## Materials and Methods

### Preparation of Oligonucleotides

DNAs (including FAM/biotin-labeled aptamers) were synthesized by Shanghai Sangon Biological Engineering Technology & Services (Shanghai, China). The BC15-31s in the animal experiment and the LNA- and 2′-*O*-MOE-modified BC15-31s were synthesized using Qingke-DNAchem-12 synthesizer according to the literature procedure using standard phosphoramidite chemistry (Huang et al., 2013, 2015; Ma et al., 2017). The standard iodine solution was replaced by phenylacetyl disulfide solution (5 g in 2 ml pyridine) in the oxidation step to complete the PS modification. Since phenylacetyl disulfide has no stereoselectivity, the synthesized oligonucleotides are racemates. Cleavage was performed in concentrated ammonia/40% methylamine water solution (1/1 vol/vol) at 65°C for 90 min. The BC15-31 variants were purified by C18 reverse high-performance liquid chromatography (XBridge^TM^ OST C18, 2.5 μm, 10 mm × 50 mm) using a linear gradient of 5 to 40% eluent A for 25 min. CH_3_CN was used as eluent A, and a solution of 0.05 M (Et_3_N)_2_CO_3_ in water was used as eluent B; the column temperature is 25°C, and the flow rate is 1.5 ml/min. Then, the isolated 5′-DMT-protected oligonucleotides were treated with 80% acetic acid at room temperature for 10 min. After neutralized with Et_3_N, the oligonucleotide solutions were desalted by Sephadex G25 column, respectively. The oligonucleotide compositions were confirmed by MALDI-TOF-MS spectrometry (Supplementary Figures 2-42).

### Cell Culture and Nanocomplex Preparation

A375 and SK-MEL-2 cells were grown in Dulbecco’s modified Eagle’s medium (M&C, Beijing, China), BcPAP, SW-480, MCF-7, MCF-7/ADR, A549, A549/Taxol, and HepG-2 cells were grown in Roswell Park Memorial Institute (RPMI)-1640 medium (M&C), supplemented with 10% fetal bovine serum (Gibco, Grand Island, United States) at 37°C and 5% CO_2_ in a humidified incubator. The DNCA and CLD lipids were dissolved in ethanol, respectively, as stock solutions (1 mM). The aptamers were dissolved in H_2_O at a concentration of 0.05 nmol/μl as stock solutions. Then, DNCA, CLD, and aptamers were diluted to appropriate concentrations with GenOpti (M&C) and sonicated for 20 min under 70°C. The nanocomplexes were obtained and diluted to a certain concentration for usage.

### Cellular Antiproliferation Assay

The CCK-8 assay was used to evaluate the cytotoxicity and the antiproliferative activity of nanocomplexes (concentration of aptamers: 25 nM). A549/Taxol (4 × 10^3^ per well), A375, SK-MEL-2, MCF-7, MCF-7/ADR, A549 cells (5 × 10^3^ per well), BcPAP, SW-480, and HepG-2 cells (1 × 10^4^ per well) were seeded into a 96-well plate and incubated with the nanocomplexes for 48 h. The group incubated with the nanocomplex of the negative control sequence (NC: 5′-TGG TGC GGT GTG GGT CGA GTG GTA TGG TATG-3′) was used as the blank. According to standard, 10 μl CCK-8 solution (Dojindo Laboratories, Beijing, China) was added to a 96-well plate. The absorbance at 450 nm was measured by a microplate reader (Molecular Devices, San Francisco, CA, United States). The relative cell viability was calculated by the equation:

(1) $$\left[\left(R_{A}-R_{E}\right)/\left(R_{B}-R_{E}\right)\right]\times 100\%$$

(*R*_*A*_, *R*_*B*_, and *R*_*E*_ are defined as the absorbance of experimental samples, untreated samples, and blank controls, respectively).

### Flow Cytometry Assay

A375 cells (4 × 10^4^ per well) were cultured in 24-well plates and proliferated for 16-24 h. FAM-labeled aptamers were used to prepare the nanocomplexes (FAM-labeled aptamers: 25 nM). The nanocomplexes and FAM-labeled aptamers were exposed to cells and incubated for 4 h, respectively. Subsequently, the cells were harvested and centrifuged at 1,000 × *g* for 3 min. The precipitates were washed by precooled phosphate-buffered saline (PBS) and filtered for a homogeneous distribution in the solution. The cellular uptake was observed on a CytoFLEX (Beckman Coulter, South Kraemer Boulevard, Brea, CA, United States) immediately.

### Confocal Microscopy Assay

A375 cells (1.4 × 10^5^ per well) were cultured at confocal observation dishes and proliferated for 16-24 h. FAM-labeled aptamers were used to prepare the nanocomplexes. The nanocomplexes and FAM-labeled aptamers were exposed to cells and incubated for 4 h, respectively. Subsequently, the culture medium was removed, followed by washing twice with PBS. Cells were stained with Hoechst 33342 (Solarbio, Beijing, China) for 20 min and washed twice with PBS and then observed under an A1Rsi confocal microscope (Nikon Instruments Inc., Tokyo, Japan). Confocal images were obtained using NIS-Elements software (Nikon Instruments Inc.).

### Sizes, Zeta Potentials, and Transmission Electron Microscopy Morphology Assay

The aptamer lipoplexes (aptamer: 10 μM) and free lipids were diluted in PBS, following filtration by a 0.22-μm filter. Then, particle sizes and ζ potentials were measured at 25°C and analyzed using dynamic light scattering (DLS) (Malvern Zetasizer Nano ZS, Worcestershire, United Kingdom). The morphology of lipoplexes was examined using transmission electron microscopy (TEM, JEM-1400Plus, 120 kV, Jeol, Tokyo, Japan). The lipoplexes were negatively stained with 1% uranyl acetate.

### Apoptosis Assay

Cellular apoptosis was quantified using an Annexin V-APC/PI Apoptosis Detection Kit (KeyGen Biotech, Nanjing, China). After treatment with nanocomplexes of BC15-31 and WW-24, the A375 cells seeded into six-well plates were harvested after 24 h of incubation. Subsequently, the cells were washed with cold PBS twice and resuspended in 500 μl of 1 × binding buffer. Next, 5 μl Annexin V-APC and 5 μl PI were added to each sample, and the cells were incubated at room temperature for 20 min in the dark. The stained samples were then determined by CytoFLEX (Beckman Coulter).

### Surface Plasmon Resonance Experiments

Surface plasmon resonance (SPR) experiments were performed on Biacore T100 (GE Healthcare, Pittsburgh, PA, United States) to know the binding kinetics of aptamers and hnRNP A1. The commercially purchased gold substrates CM5 (Thermo Fisher, Waltham, MA, United States) were used as the SPR sensor chips. The reagents N-hydroxysuccinimide (NHS) and N-ethyl-N’-(3-diethylaminopropyl) carbodiimide hydrochloride (EDC) were used to activate the sensor chips, and recombined protein hnRNP A1 (ProSpec-Tany TechnoGene Ltd., Ness-Ziona, Israel) was linked on the sensor chips. Then, the unreacted active carboxyl intermediate was blocked with ethanolamine. The running buffer for immobilization and sample analysis was 10 mM phosphate-buffered saline (138 mM NaCl, 2.7 mM KCl, 10 mM Na_2_HPO_4_, 1.76 mM KH_2_PO_4_, 0.05% Tween-20), pH 7.4, at 25°C. Every aptamer solution was sequentially injected over the sensor surface for 3 min at 30 μl/min and 5 min dissociation time. For each sample, six aptamer concentrations were injected by serially diluting samples from 0.39 to 50 nM and a blank sample only containing running buffer for referencing. The unbound aptamer was removed by treatment with 4.0 M NaCl aqueous, and the chip was primed before use. The raw data were processed and analyzed to determine the binding constant for each oligonucleotide. In order to correct refractive index changes and instrument noise, the response data from the control surface were subtracted from the responses obtained from the reaction surface using BIA evaluation 4.1 software.

### Serum Stability of PS-Modified Aptamers

Aptamer (140 pmol) was diluted to 56 μl with PBS. The 56-μl sample was divided equally into seven tubes, and 2 μl fetal bovine serum was added into six tubes, respectively. One tube was added with 2 μl PBS as the blank group. The solutions were incubated at 37°C for 0, 0.5, 1, 2, 4, and 8 h. Each tube was removed and inactivated at 95°C for 5 min, and then placed in a –80°C refrigerator for detection. All samples were resolved with 20% polyacrylamide denaturing (7 M urea) gel electrophoresis and visualized by staining with SYBR-Gold and quantified by using a ChemiDoc XRS highly sensitive chemiluminescence gel imaging analyzer (Bio-Rad, Hercules, CA, United States).

### Target Fishing Experiment

Biolayer interferometry (BLI) was used to investigate the target of WW-24. WW-24 was biotinylated at the 5′-terminal (Sangon, China). A375 cell lysate was prepared by the nuclear protein extraction kit (Solarbio, Beijing, China). Biotin-WW-24 was diluted to 5 μg/μl and loaded to six SSA sensors for 420 s, and the sensors were infiltrated in PBS for 60 s and then put into A375 cell lysate for fishing. We can see a strong association only in the experiment group and nearly no association in the blank group. After fishing for 300 s, the sensors were inserted into 0.1% formic acid solution for the wash. The experiment was repeated 100 times, and the formic acid solution of the experiment and blank groups was gathered up, respectively, for MS analysis.

### Animal Experiments of Antitumor Efficiency

The committee approved all animal experiments for animal research of Peking University (no. LA2017194). In addition, all of the operations on animals conformed to the National Institutes of Health Guide for the Care and Use of Laboratory Animals (NIH Publications no. 8023, revised 1978). Specific pathogen-free (SPF)-grade female BALB/c nude mice (3-4 weeks) were obtained from Wantonglihua (China) and kept at the Department of Laboratory Animal Science, Peking University Health Science Center.

A375 cells (1.6 × 10^6^) were subcutaneously inoculated into the mice. When the tumor volumes reached 50-80 mm^3^ in size, the mice were randomly divided into four groups, which were treated with GenOpti (free solvent, blank), NC/Mix nanocomplex, BC15-31/Mix nanocomplex, and WW-24/Mix nanocomplex (2.0 mg/kg) by peritumoral injection, respectively. The mice (*n* = 5) received the treatment at days 1, 3, 5, 7, and 9. The tumor volumes (mm^3^) were calculated by equations:

(2) Volume=length×width2×0.5

(3) Re=Volume/Volume1

[Re, Volume, and Volume 1 were defined as the relative tumor volume, practical tumor volume, and original (day 1) tumor volume, respectively.]

### Biodistribution Assay *in vivo*

Female BALB/c nude mice (3-4 weeks) bore xenografted A375 cells (1.6 × 10^6^) subcutaneously under the right arm. When the tumor volume reached about 1,200 mm^3^, different formulations consisting of Cy7-labeled aptamers, DNCA, CLD, and PEG-DSPE were injected by the peritumoral an aptamer concentration of 1.5 mg/kg. The images were taken at 1, 2, 4, 8, 12, and 24 h after injection utilizing the IVIS Spectrum *in vivo* imaging system (PerkinElmer, Shanghai, China). The measurements were performed at 745 nm excitation wavelength and 800 nm emission wavelength. The mice were sacrificed at 24 h time points, and the tumors and organs were then isolated for the *ex vivo* distribution assay.

### Statistical Analysis

All data in this study are average values, and error bars represent standard deviations. Two groups of independent data were analyzed by *t*-test, and one-way ANOVA analyzed multiple groups of independent data. Significance of *P*-value: n.s. means *P* > 0.05; ^∗^ means *P* < 0.05; ^∗∗^ means *P* < 0.01; ^∗∗∗^ means *P* < 0.001. All statistical analyses were performed using GraphPad Prism 8.0 software.

## Results

### Design of Liposome and Screening of Modification Sites

The PS modification of DNA can influence the properties of oligonucleotide in various ways, such as serum stability and protein-binding capacity (Hu et al., 2015). An excess of PS modification sites will enhance the protein-binding ability, but it may decrease the special binding with its target protein simultaneously, which indicated that the PS backbone should only be modified at necessary sites; this is the key to improving its specific target binding affinity. LNA imposes a conformational restriction to adopt an N-type sugar puckering (Koshkin et al., 1998). The LNA modification strategy could improve the properties of the oligonucleotides, thereby increasing their potential for therapeutic purposes. LNA modification could improve the biological stability and target affinity of aptamers (Schmidt et al., 2004; Lebars et al., 2007). 2′-*O*-MOE is usually used as the second-generation modification strategy of antisense nucleic acid to improve its pharmacokinetic properties, reduce the immunostimulatory side effects caused by PS modification, and improve its affinity (Manoharan, 1999).

**FIGURE 1:**
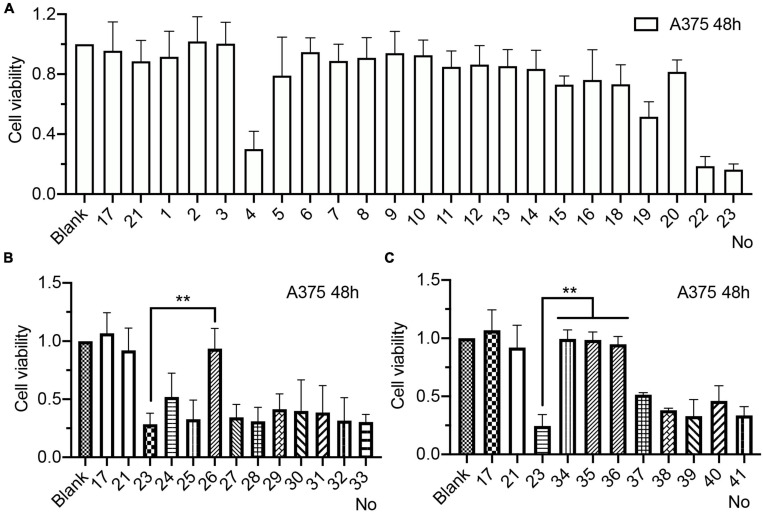
Antiproliferation activity of chemical modified BC15-31s (DNCA/CLD/nt: 4:1:1.25 nM; 48 h) in A375 cell line. **(A)** Proliferation inhibitory effect of aptamer with PS modification. **(B)** Proliferation inhibitory effect of aptamer with LNA modification. **(C)** Proliferation inhibitory effect of aptamer with 2′-*O*-MOE modification. Numbers “1,” “2,” “3,” etc. correspond to the serial number in Table 1. Experimented with CCK-8 reagent, **P < 0.01.

Three consecutive positions of PS-modified BC15-31s (WW-1∼WW-20) were synthesized (Table 1), while mixed DNCA/CLD(Mix) was used to encapsulate the BC15-31s at an empirical molecular ratio (DNCA:CLD:aptamer = 124:31:1). We treated the A375 cell at a concentration of 25 nM to investigate the antiproliferation. After 48 h, WW-4 and WW-19 (Table 1) showed good antiproliferation activity (Figure 1A). Based on that of WW-4 and WW-19, two sequences WW-23 and WW-24 were designed and synthesized (Table 1), which showed excellent antitumor activity. We discerned that the concentration of 25 nM is too low for an unmodified aptamer BC15-31 to exert an ideal antitumor activity. The antiproliferation activity of the NC group clearly revealed that the cytotoxicity of mixed lipids is very low at this concentration. Ten LNA-T-modified sequence, 24-L1∼24-L10, as well as eight 2′-*O*-MOE-modified sequences, 24-M1∼24-M8 (Table 1), were further designed and synthesized. However, no individual showed significant biological improvements compared with WW-24 (Figures 1B,C). Further optimization of the preparation DNCA:CLD:aptamer = 31:31:1 could give identical biological effect with WW-23 and WW-24 (Supplementary Figure 1).

**TABLE 1:** The sequences of phosphothioated aptamer BC15-31s.

**No.**	**Name**	**Sequence (5′-3′)**
1	WW-1	T^∗^G^∗^T^∗^GGCGAGGTAGGTGGGGTGT GTGTGTATC
2	WW-2	TGTG^*^ G^*^ C^*^ GAGGTAGGTGGGGTGTGTGTGTATC
3	WW-3	TGTGGC^*^ G^*^ A^*^ GGTAGGTGGGGTGTGTGTGTATC
4	WW-4	TGTGGCGAG^*^ G^*^ T^*^ AGGTGGGGTGTGTGTGTATC
5	WW-5	TGTGGCGAGGTA^*^ G^*^ G^*^ TGGGGTGTGTGTGTATC
6	WW-6	TGTGGCGAGGTAGGT^*^ G^*^ G^*^ GGTGTGTGTGTATC
7	WW-7	TGTGGCGAGGTAGGTGGG^*^ G^*^ T^*^ GTGTGTGTATC
8	WW-8	TGTGGCGAGGTAGGTGGGGTG^*^ T^*^ G^*^ TGTGTATC
9	WW-9	TGTGGCGAGGTAGGTGGGGTGTGT^*^ G^*^ T^*^ GTATC
10	WW-10	TGTGGCGAGGTAGGTGGGGTGTGTGTG^*^ T^*^ A^*^ TC
11	WW-11	TGTGGCGAGGTAGGTGGGGTGTGTGTGT^*^ A^*^ T^*^ C
12	WW-12	T^*^ G^*^ T^*^ G^*^ GCGAGGTAGGTGGGGTGTGTGTGTATC
13	WW-13	TGTGGCGAGG^*^ T^*^ A^*^ G^*^ GTGGGGTGTGTGTGTATC
14	WW-14	TGTGGCGAGGTAGGTG^*^ G^*^ G^*^ G^*^ TGTGTGTGTATC
15	WW-15	TGTGGCGAGGTAGGTGGGGTGT^*^ G^*^ T^*^ G^*^ TGTATC
16	WW-16	TGTGGCGAGGTAGGTGGGGTGTGTGTG^*^ T^*^ A^*^ T^*^ C
17	BC15-31	TGTGGCGAGGTAGGTGGGGTGTGTGTGTATC
18	WW-18	T^*^ G^*^ T^*^ GGCGAGGTAGGTGGGGTGTGTGTGT^*^ A^*^ T^*^ C
19	WW-19	T^*^ G^*^ T^*^ GGCGAGGTA^*^ G^*^ G^*^ TGGGGTGTGTGTGT^*^ A^*^ T^*^ C
20	WW-20	T^*^ G^*^ T^*^ G^*^ G^*^ C^*^ G^*^ A^*^ G^*^ G^*^ T^*^ A^*^ G^*^ G^*^ T^*^ G^*^ G^*^ G ^*^ G^*^ T^*^ G^*^ T^*^ G^*^ T^*^ G^*^ T^*^ G^*^ T^*^ A^*^ T^*^ C
21	NC	TGGTGCGGTGTGGGTCGAGTGGTATGGTATG
22	WW-23	T^*^ G^*^ T^*^ GGCGAG^*^ G^*^ T^*^ AGGTGGGGTGTGTGTGT^*^ A^*^ T^*^ C
23	WW-24	T^*^ G^*^ T^*^ GGCGAG^*^ G^*^ T^*^ A^*^ G^*^ G^*^ TG GGGTGTGTGTGT^*^ A^*^ T^*^ C
24	24-L1	T_LNA_^*^ G^*^ T^*^ GGCGAG^*^ G^*^ T^*^ A^*^ G^*^ G^*^ TGGGG TGTGTGTGT^*^ A^*^ T^*^ C
25	24-L2	T^*^ G^*^ T_LNA_^*^ GGCGAG^*^ G^*^ T^*^ A^*^ G^*^ G^*^ TGGGGT GTGTGTGT^*^ A^*^ T^*^ C
26	24-L3	T^*^ G^*^ T^*^ GGCGAG^*^ G^*^ T_LNA_^*^ A^*^ G^*^ G^*^ TGGGG TGTGTGTGT^*^ A^*^ T^*^ C
27	24-L4	T^*^ G^*^ T^*^ GGCGAG^*^ G^*^ T^*^ A^*^ G^*^ G^*^ T_*LNA*_GGGGT GTGTGTGT^*^ A^*^ T^*^ C
28	24-L5	T^*^ G^*^ T^*^ GGCGAG^*^ G^*^ T^*^ A^*^ G^*^ G^*^ TGGGGT_*LNA*_GT GTGTGT^*^ A^*^ T^*^ C
29	24-L6	T^*^ G^*^ T^*^ GGCGAG^*^ G^*^ T^*^ A^*^ G^*^ G^*^ TGGGGTGT_*LNA*_G TGTGT^*^ A^*^ T^*^ C
30	24-L7	T^*^ G^*^ T^*^ GGCGAG^*^ G^*^ T^*^ A^*^ G^*^ G^*^ TGGGGTGTGT_*LNA*_G TGT^*^ A^*^ T^*^ C
31	24-L8	T^*^ G^*^ T^*^ GGCGAG^*^ G^*^ T^*^ A^*^ G^*^ G^*^ TGGGG TGTGTGT_*LNA*_GT^*^ A^*^ T^*^ C
32	24-L9	T^*^ G^*^ T^*^ GGCGAG^*^ G^*^ T^*^ A^*^ G^*^ G^*^ TGGGG TGTGTGTGT_LNA_^*^ A^*^ T^*^ C
33	24-L10	T^*^ G^*^ T^*^ GGCGAG^*^ G^*^ T^*^ A^*^ G^*^ G^*^ TGGG GTGTGTGTGT^*^ A^*^ T_LNA_^*^ C
34	24-M1	T_MOE_^*^ G_MOE_^*^ T^*^ GG_*MOE*_C_*MOE*_GAG^*^ G^*^ T^*^ A^*^ G^*^ G^*^ TGGGGT GTGTGTGT^*^ A^*^ T^*^ C
35	24-M2	T^*^ G^*^ T_MOE_^*^ G_*MOE*_GCG_*MOE*_A_*MOE*_G^*^ G^*^ T^*^ A^*^ G^*^ G^*^ TGGGGT GTGTGTGT^*^ A^*^ T^*^ C
36	24-M3	T^*^ G^*^ T^*^ GGCGAG_MOE_^*^ G_MOE_^*^ T^*^ A^*^ G_MOE_^*^ G_MOE_^*^ TGGGGTG TGTGTGT^*^ A^*^ T^*^ C
37	24-M4	T^*^ G^*^ T^*^ GGCGAG^*^ G^*^ T_MOE_^*^ A_MOE_^*^ G^*^ G^*^ T_*MOE*_G_*MOE*_G GGTGTGTGTGT^*^ A^*^ T^*^ C
38	24-M5	T^*^ G^*^ T^*^ GGCGAG^*^ G^*^ T^*^ A^*^ G^*^ G^*^ TGG_*MOE*_G_*MOE*_GTG_*MOE*_T _*MOE*_GTGTGT^*^ A^*^ T^*^ C
39	24-M6	T^*^ G^*^ T^*^ GGCGAG^*^ G^*^ T^*^ A^*^ G^*^ G^*^ TGGGG_*MOE*_T_*MOE*_GT G_*MOE*_T_*MOE*_GTGT^*^ A^*^ T^*^ C
40	24-M7	T^*^ G^*^ T^*^ GGCGAG^*^ G^*^ T^*^ A^*^ G^*^ G^*^ TGGGGTGTGTG_*MOE*_T _*MOE*_GT^*^ A^*^_MOE_T^*^_MOE_C
41	24-M8	T^*^ G^*^ T^*^ GGCGAG^*^ G^*^ T^*^ A^*^ G^*^ G^*^ TGGGGTGTGTGTG_*MOE*_T ^*^_MOE_A^*^ T^*^ C

*“^∗^” represents phosphorothioate modification site; “T_*LNA*_” represents LNA-T modification; “B_*MOE*_” represents 2′-MOE modification.*

**FIGURE 2:**
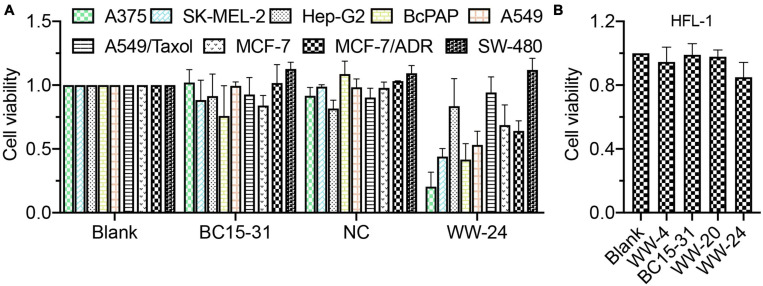
The cell viability of various cell lines with lipid/aptamer nanocomplexes (DNCA/CLD/nt: 1:1:1, aptamers: 25 nM, 48 h). **(A)** The proliferation inhibition of aptamers on tumor cell lines. **(B)** The proliferation inhibition of aptamers on human normal cell line. Experimented with CCK-8 reagent.

As hnRNPs were overexpressed in most tumor cells, MCF-7, M/A, A549, A/T, HepG2, SK-MEL-2, BcPAP, SW480, and A375 cell lines were treated with WW-24/Mix at 25 nM (Figure 2A), which showed strong antiproliferation effect on the A375 cell line; moderate on the SK-MEL-2, BcPAP, and A549 cell lines; and almost no activity on the others. On human normal cell line HFL-1, the following preparations (Figure 2B) were found to be almost non-toxic.

### Characterization of DNCA/CLD/aptamer Liposome

**FIGURE 3:**
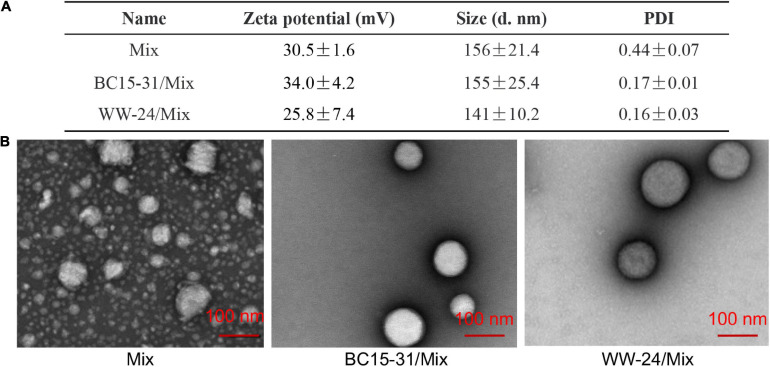
Characterization of DNCA/CLD/aptamer liposomes. **(A)** The size, zeta potential, and PDI of the nanocomplexes. **(B)** TEM result of DNCA/CLD/aptamer nanoparticles (DNCA/CLD/nt: 1/1/1; aptamers: 10 μM).

Dynamic Light Scattering (DLS) was used to investigate the particle size and zeta potentials of BC15-31/Mix and WW-24/Mix. The results (Figure 3A) showed that were around 150 nM and 34 ± 4.2 and 25.8 ± 7.4 mV, respectively. A transmission electron microscope (TEM) was then used to verify the size of liposomes again. Without aptamers, the surface of liposomes will shrink (Figure 3B). The WW-24/Mix nanoparticles can form uniform spherical nanoparticles with smooth edges. In summary, the BC15-31s/Mix can form spherical structures with sizes between 130 and ∼180 nm, and the surfaces were positively charged.

### Transmembrane Assay of DNCA/CLD Enhances Aptamers

**FIGURE 4:**
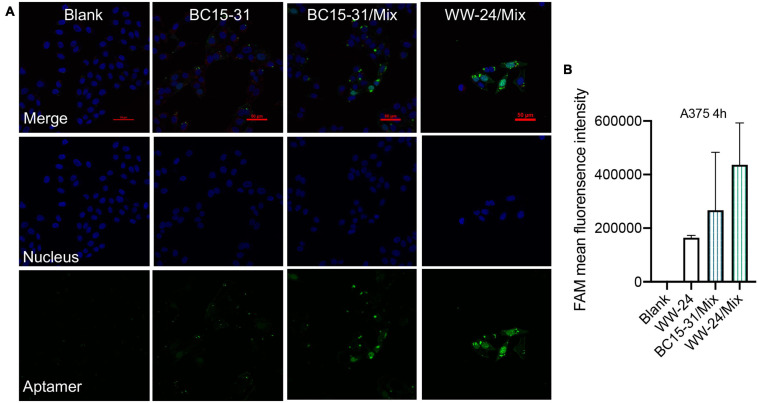
Liposomes improve transmembrane ability of aptamers. **(A)** Confocal analysis of lipid/aptamer nanocomplex permeation (FAM-aptamers, 25 nM). The cells were observed at 6 h after incubation with blank, FAM-BC15-31, FAM-BC15-31/Mix, and FAM-WW-24/Mix nanocomplexes, in which the aptamer and nucleus were visible by green and blue colors. **(B)** Cellular uptake of lipid/aptamer nanocomplex permeation (FAM-aptamers, 25 nM, *n* = 3) at 4 h. All data in this study are average values, and error bars represent standard deviations.

Confocal analysis was performed to investigate the subcellular distribution of BC15-31/Mix and WW-24/Mix (Figure 4A). The results showed that the nanoparticles could be delivered into cells with high efficiency. Without a DNCA/CLD encapsulation, the BC15-31 aptamer had almost no fluorescence at 6 h. Compared with BC15-31/Mix, WW-24/Mix was significantly enriched in cells after 6 h; this may be attributed to the better stability by PS modification. Both BC15-31 and WW-24 preferred to go into the nucleus. Flow cytometry experiments showed that, compared with the non-encapsulated BC15-31 (Figure 4B), BC15-31s/Mix had improved ability to deliver the aptamers into cells. Specifically, WW-24 was more stable in cells compared with BC15-31.

### Phosphorothioate Modification Promotes BC15-31’s Stability and Target Affinity

**FIGURE 5:**
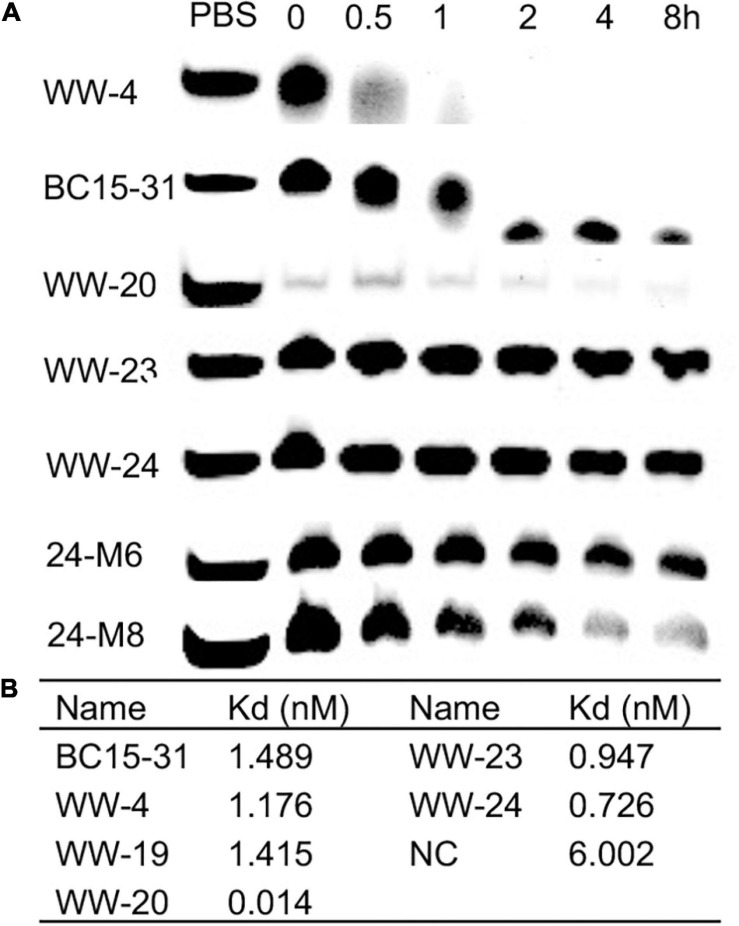
**(A)** Serum stability of representative PS-modified BC15-31s (20% FBS; 20 pmol aptamer). **(B)** Binding affinity of PS-modified BC15-31s to hnRNP A1.

In 1967, Eckstein (1967) (De Clercq et al., 1969) first reported the strategy of PS modification to enhance the ribonucleases (RNase) resistance of RNA. Some of the BC15-31s with good anti-proliferation activities were selected, which were incubated in 20% fetal bovine serum. PAGE results showed that, compared with BC15-31, the serum stability of 3′-terminal modified aptamers (including WW-19, WW-20, WW-23, WW-24) were dramatically improved (Figure 5A).

SPR experiments were performed to investigate the target affinity of BC15-31s. We immobilized the protein hnRNP A1 instead of aptamers on the chip, using the amino coupling strategy. BC15-31s that showed good antitumor activities were selected to investigate the target affinity. The results showed that the target affinities of all modified BC15-31s were improved compared with BC15-31 (Figure 5B). All site PS-modified sequence WW-20 showed the strongest binding ability (14 pM), which was very hard to regenerate. It might be the reason why it does not show ideal antitumor activity due to non-special binding.

### Mix-WW-24 Promotes the Process of Apoptosis

**FIGURE 6:**
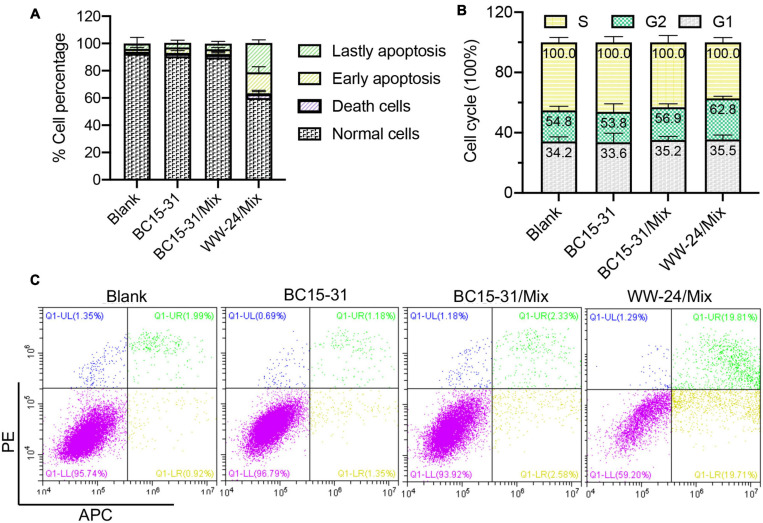
**(A)** The apoptosis analysis of BC15-31/Mix and WW-24/Mix (25 nM, 24 h, *n* = 3). **(B)** The cell cycle of BC15-31/Mix and WW-24/Mix (25 nM, 24 h, *n* = 3). **(C)** The typical picture of apoptosis. All data in this study are average values, and error bars represent standard deviations.

Different from cell necrosis, apoptosis is an active process involving the activation, expression, and regulation of a series of genes (Reed, 2000). The apoptosis effects of BC15-31/Mix and WW-24/Mix on A375 cell were investigated. The results (Figure 6A) showed that, compared with BC15-31, WW-24 can promote early and late apoptosis (∼35%), while 7% of the cell cycle is blocked in the G2/M phase (Figure 6B). The typical picture of apoptosis were shown here (Figure 6C).

### Target Fishing Experiment

BLI was used to “fish” the target of WW-24 from the A375 cell. Nuclear protein was extracted, since the aptamers were mainly distributed in the nucleus. We coupled biotin to the 5′-terminal of WW-24 and loaded it to the SA sensors and then put the sensors into the nuclear protein extraction solutions. Table 2 showed the results of our experiment. Here, a strong combination was observed compared with the blank group. After that, the sensors were pulled out and inserted into formic acid solutions to desorb the binding proteins. Thus, the binding proteins will be enriched in formic acid solutions, and then MS analysis was performed.

**TABLE 2:** Target protein of aptamer WW-24.

**Protein**	**PBS**	**NC**	**WW-24**
	**PSMs**	**PSMs**	**PSMs**
Heterogeneous nuclear ribonucleoprotein A1	25	111	281
Heterogeneous nuclear ribonucleoproteins A2/B1	24	63	229
Heterogeneous nuclear ribonucleoprotein U	-	79	81
Desmoplakin	42	28	85
Heterogeneous nuclear ribonucleoprotein D0	8	55	39

The results showed that the abundance of hnRNPs in the experimental group was significantly higher than that of the blank group. We concluded that the most different proteins, hnRNP A2/B1 and hnRNPA1, were the key target proteins related to the proliferation activity of WW-24. The specificity of the aptamers is not as good as that of antibodies because the hydrophobic interaction and the electrostatic interaction made outstanding contributions to the stable 3D structure formation of antibody, and this did not play an essential role in the structure formation of the aptamers. In addition, the aptamers are smaller than antibodies, which means that the aptamers may bind to similar substructures of different proteins and that hnRNP A1 has the same domain structure as hnRNP A2/B1 (Dreyfuss et al., 2002).

### Biodistribution

**FIGURE 7:**
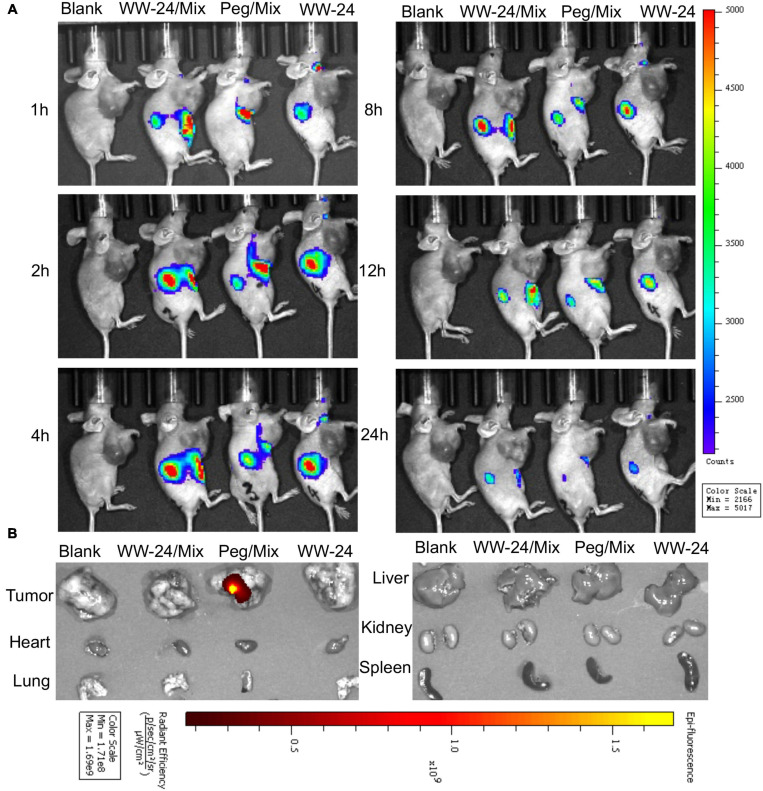
Distribution of nanoparticles of WW-24/Mix with 1% PEG-2000 *in vivo* by peritumoral injection. The biodistribution of Cy7-labeled WW-24 was observed by an IVIS spectrum *in vivo* imaging system at the excitation wavelength of 745 nm and emission wavelength of 800 nm. **(A)** Typical *in vivo* imaging at 1, 2, 4, 8, 12, and 24 h (the black arrows show the tumor tissues). **(B)** Imaging of organs and tumors at 48 h excised from BALB/c nude mice *ex vivo*.

In order to extend the metabolism time of the aptamers *in vivo* and enhance the stability of the nanoparticles, 1% PEG was introduced into the liposome system (Peg/Mix). After the peritumoral injection, we determined that there was almost no difference in tissue distribution; however, when the mice were dissected after 48 h, the PEG group showed a better tumor accumulation (Figure 7).

### Antitumor Efficiency and Toxicity of WW-24/liposome *in vivo*

**FIGURE 8:**
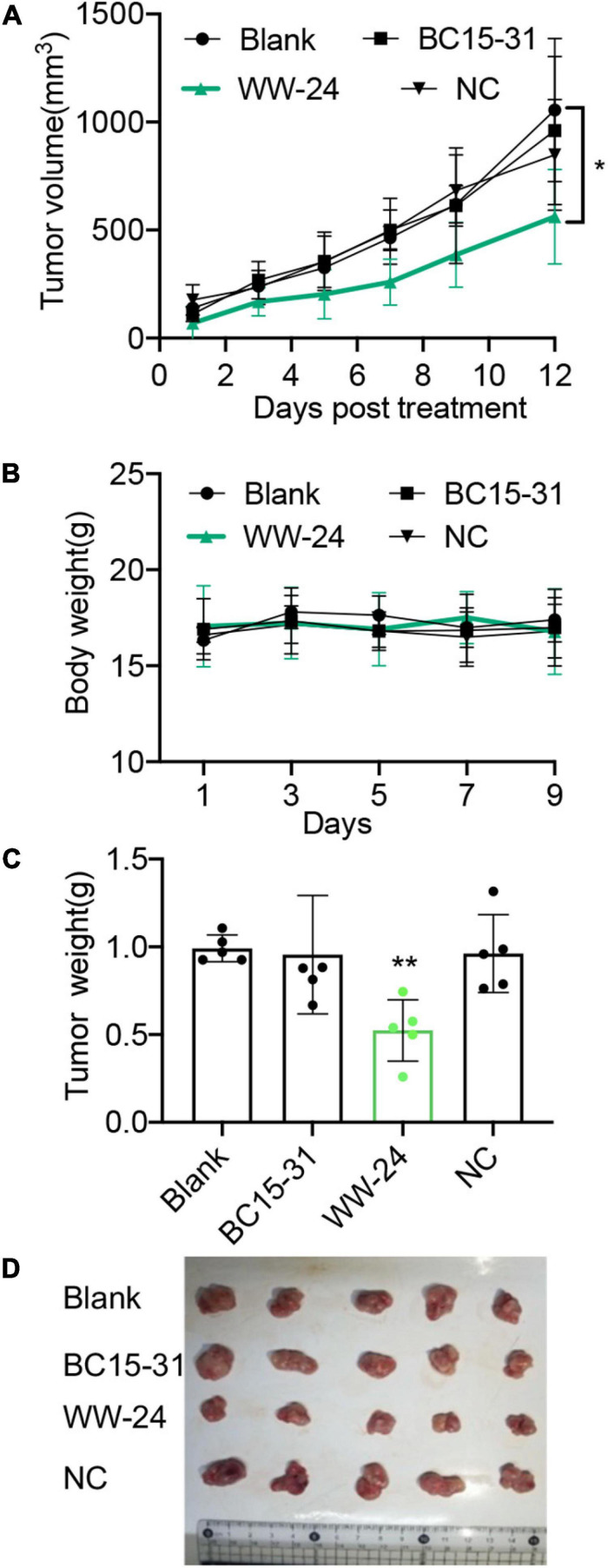
Antitumor efficiency and biosafety of WW-24/Mix nanocomplexes *in vivo*. A375 cells were inoculated into BALB/c mice. When the tumors had reached 50-80 mm^3^, the mice were treated with GenOpti (blank) or aptamers/Mix by peritumoral injection (2 mg/kg). **(A)** Tumor volumes were measured and calculated (*n* = 5, *P < 0.05). **(B)** Body weights of mice treated with nanocomplexes (*n* = 5). **(C)** The comparison of each group tumor weight and the bar chart. Statistical analysis was carried out utilizing multiple *t*-test one per row, and statistical significance was corrected using the Holm-Sidak method for *post hoc* analysis **(A/B)**. **P < 0.01. **(D)** The picture of each group tumor. All data in this study are average values, and error bars represent standard deviations.

Subcutaneous A375 tumors in the BALB/c mice model were established to evaluate the antitumor efficiency and safety *in vivo*. Twenty mice were divided into four groups (*n* = 5); these groups were then treated with GenOpti (blank), NC/Mix, BC15-31/Mix, and WW-24/Mix by peritumoral injection (2 mg/kg), respectively. The results showed that the tumor of the WW-24/Mix group was relatively smaller than those of the other groups (Figure 8A) after 12 days (*P* < 0.05). There was no difference in the body weights between the four groups, and all 20 mice survived for 12 days (Figure 8B). After 12 days, the tumors of all the mice were taken out and weighed. We found that the tumors of the mice in the WW-24/Mix group were significantly smaller (*P* < 0.01) than those of the other three groups (Figures 8C,D).

## Discussion

Similar to monoclonal antibodies, aptamers can specifically recognize and bind targets. Therefore, aptamers have many applications in diagnosis and therapy, such as biosensors, targeted therapy, and target inhibitors. In terms of disease treatment, aptamers can activate target receptors to affect their biological functions or serve as carriers for drug delivery to target cells or tissues. Exogenous nucleic acid drugs need to overcome multiple obstacles in order to enter the body to work (Singh et al., 2018).

Because of the limitations encountered in the delivery of nucleic acid drugs *in vivo*, various measures have been applied to solve the above problems. The more important ones are the chemical modification (Adachi and Nakamura, 2019) of nucleotides and the application of delivery systems (Baillet et al., 2018). To improve the stability of nucleic acid molecules and reduce their immunogenicity, the development of delivery system technology has made it possible to prevent nucleic acid drugs from being degraded by nucleases while improving the efficiency of their entry into cells.

The serum stability and the target affinity of aptamer BC15-31 were improved by partial PS, LNA-T, and 2′-*O*-MOE modification. A sequence WW-24 showed excellent antiproliferation activity on the A375 cell line at low concentrations (25 nm, ∼85%). In addition, the cytidinyl lipid DNCA and cationic lipid CLD were mixed at the optimal ratio to the single nucleotide(nt) (DNCA:CLD:nt = 1:1:1) as a low-toxic vector that can deliver WW-24 and reduce the dose to 25 nM. WW-24 can promote the apoptotic transformation of cancer cells in the late stage and has an obvious influence on the cell cycle. The fishing experiment showed that WW-24 can specifically bind to hnRNP A1 and hnRNP A2/B1. An intratumoral injection was used to act on the periphery of the tumor directly. It has not been confirmed that the EPR effect enables the drug to target tumor cells for a long time. Also, the pathway and mechanism of aptamer entry are not yet clear, and the binding mode of the target is also a direction that needs to be explored in the future. Although flow cytometry experiments have confirmed the phenomenon of apoptosis, the specific mechanism is still unknown. Overall, as an anticancer agent, WW-24/Mix has excellent application prospects. This provided a new idea for drug development based on aptamers.

## Data Availability Statement

The original contributions presented in the study are included in the article/Supplementary Material, further inquiries can be directed to the corresponding author.

## Ethics Statement

The animal study was reviewed and approved by The Committee for Animal Research of Peking University (no. LA2017194).

## Author Contributions

ZY conceived the project. SW and JW designed the experiment. JW performed the main experiments, analyzed the data, and reviewed the literatures and edited the manuscript. SW was responsible for most of the synthesis of the phosphothioated aptamers, performed the experiments, and wrote the original draft. XL was responsible for synthesizing the 2′-*O*-MOE-modified aptamers. QZ, JY, YM, and ZG provided experimental support. All authors contributed to the article and approved the submitted version.

## Conflict of Interest

The authors declare that the research was conducted in the absence of any commercial or financial relationships that could be construed as a potential conflict of interest.

## Supplementary Material

Supplementary Figure 1 The antiproliferation activity of WW-24 encapsulated in different ratios of DNCA/CLD upon A375 (25 nM).

Supplementary Figure 2-42 The ESI-MS of 41 aptamers.

Supplementary Figure 43-49 The gel pattern of Figure 5A.
