# Supplementary material for: Selective Anti-melanoma Effect of Phosphothioated Aptamer Encapsulated by Neutral Cytidinyl/Cationic Lipids
Source: Front Cell Dev Biol. 2021 Jun 28;9:660233. doi: 10.3389/fcell.2021.660233 (PMC8273494; doi:10.3389/fcell.2021.660233)
Supplement: Supplementary Figure 1 — The antiproliferation activity of WW-24 encapsulated in different ratios of DNCA/CLD upon A375 (25 nM). [file Data_Sheet_1.docx]

Supplementary Material

## Supplementary Figures

**Supplementary Figure 1.** The antiproliferation activity of WW-24 encapsulated in different ratios of DNCA/CLD upon A375 (25 nM).


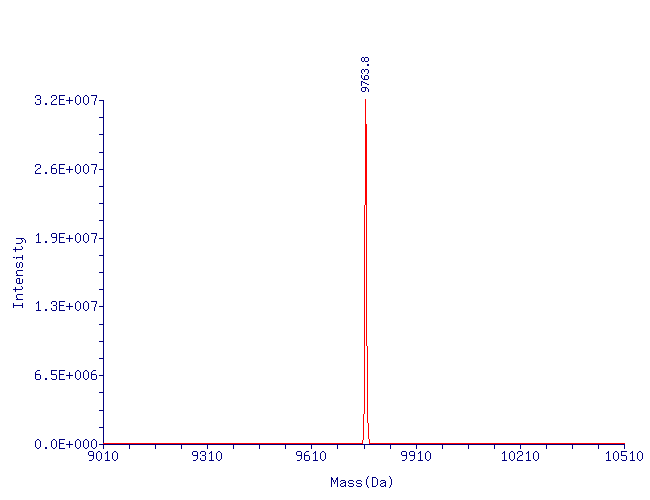

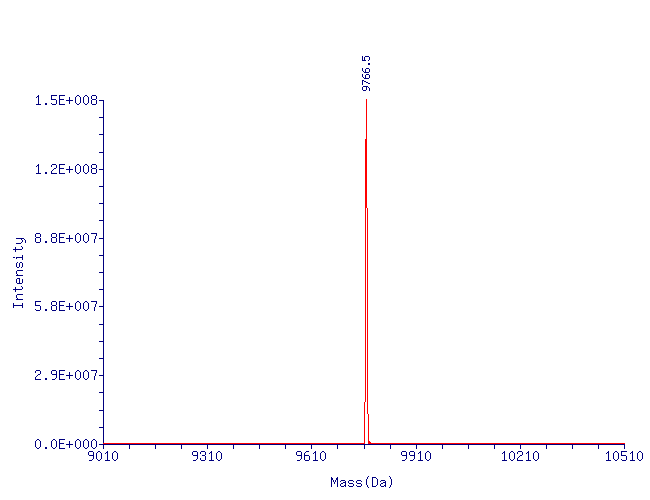


**Figure 2.** The ESI-MS of BC15-31 strand **Figure 3.** The ESI-MS of NC strand

(Calcd: 9765) (Calcd: 9765)


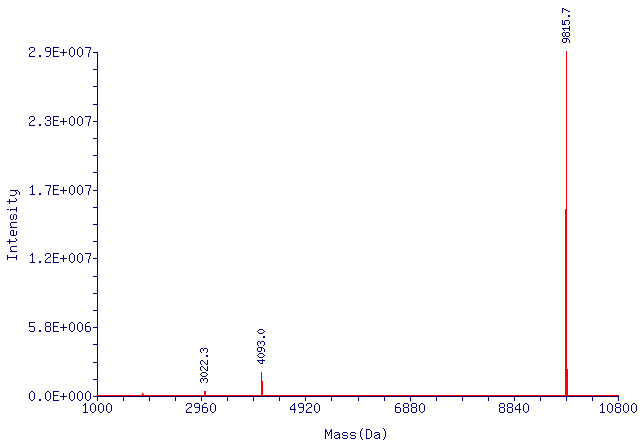

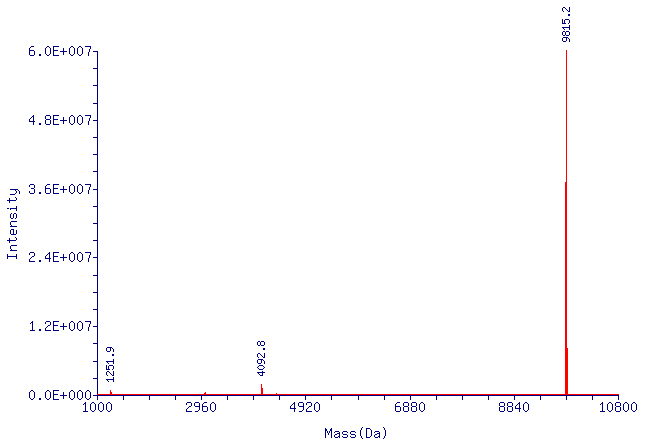


**Figure 4.** The ESI-MS of WW-1 strand **Figure 5.** The ESI-MS of WW-2 strand

(Calcd: 9813) (Calcd: 9813)


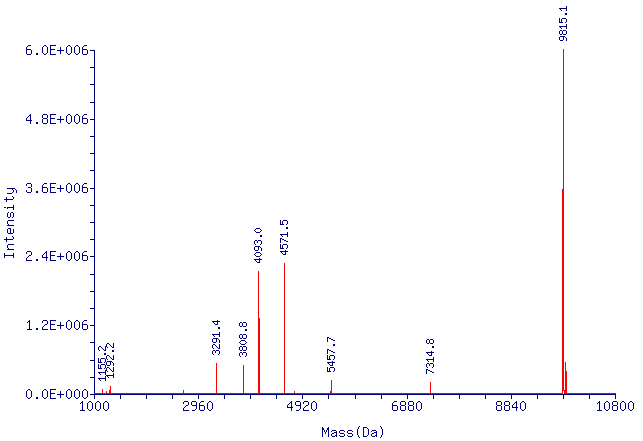

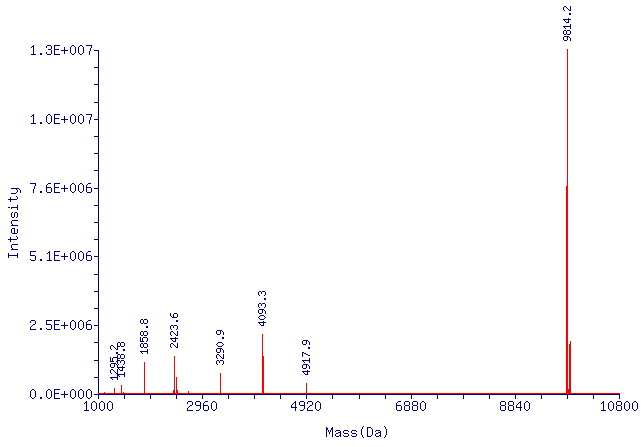


**Figure 6.** The ESI-MS of WW-3 strand **Figure 7.** The ESI-MS of WW-4 strand

(Calcd: 9813) (Calcd: 9813)


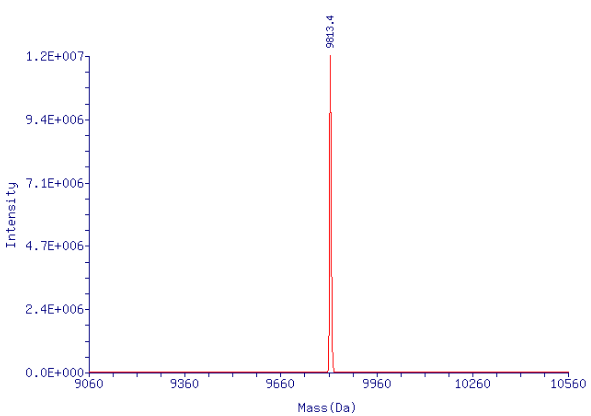

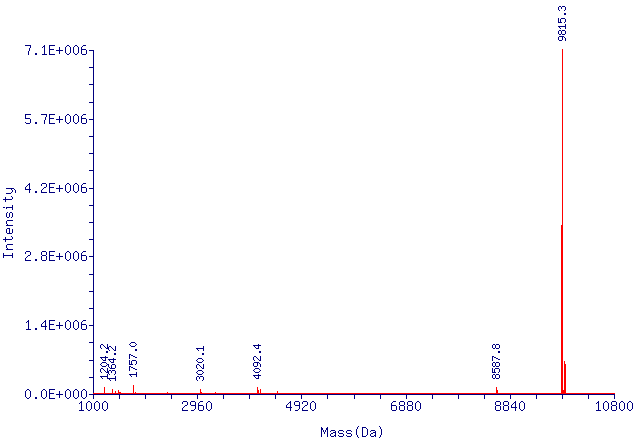


**Figure 8.** The ESI-MS of WW-5 strand **Figure 9.** The ESI-MS of WW-6 strand

(Calcd: 9813) (Calcd: 9813)


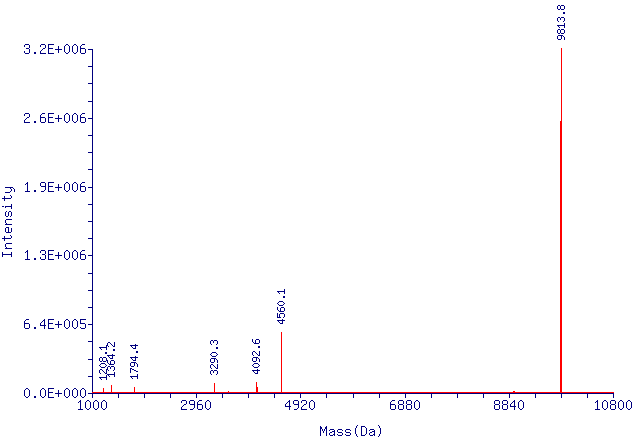

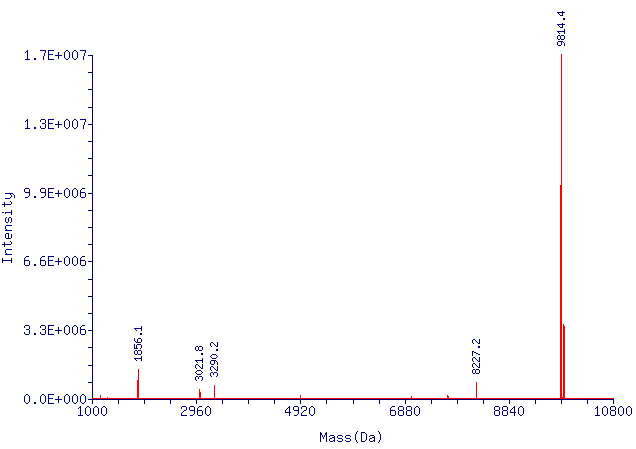


**Figure 10.** The ESI-MS of WW-7 strand **Figure 11.** The ESI-MS of WW-8 strand

(Calcd: 9813) (Calcd: 9813)


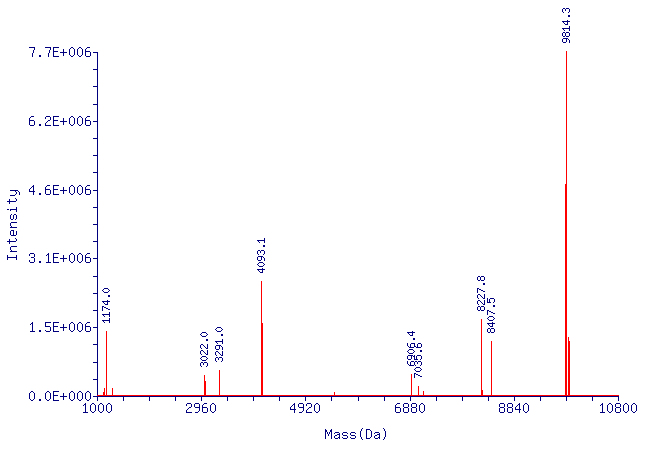

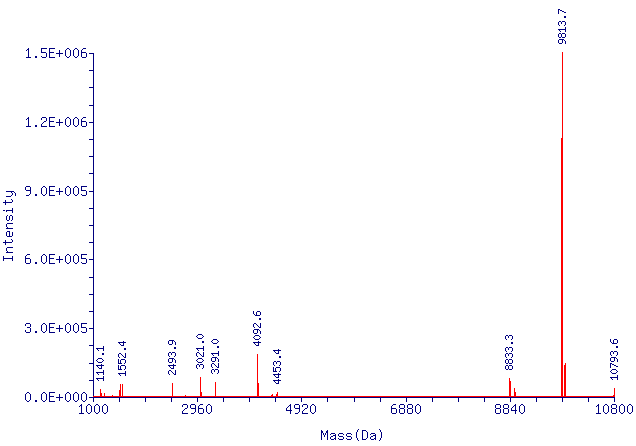


**Figure 12.** The ESI-MS of WW-9 strand **Figure 13.** The ESI-MS of WW-10 strand

(Calcd: 9813) (Calcd: 9813)


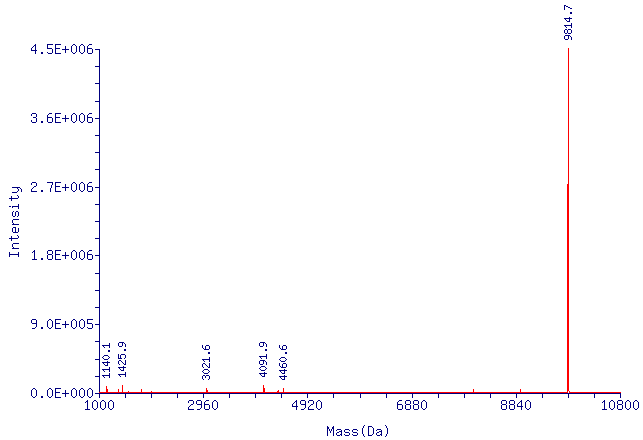

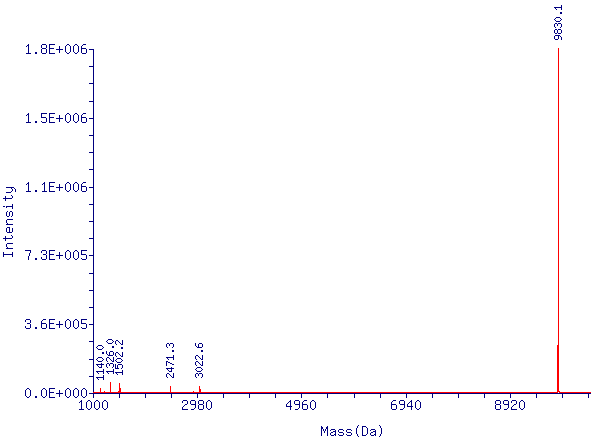


**Figure 14.** The ESI-MS of WW-11 strand **Figure 15.** The ESI-MS of WW-12 strand

(Calcd: 9813) (Calcd: 9829)


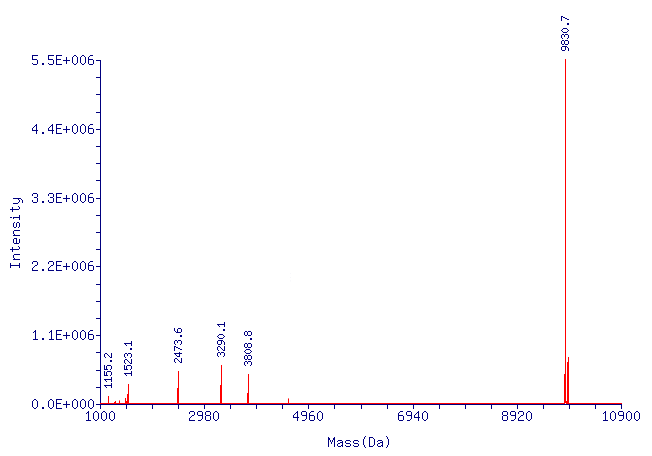

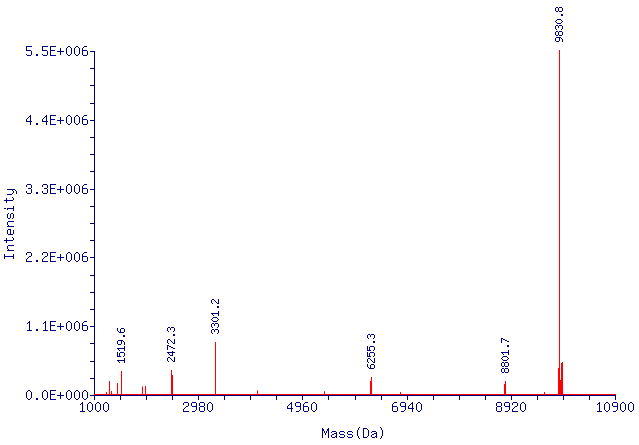


**Figure 16.** The ESI-MS of WW-13 strand **Figure 17.** The ESI-MS of WW-14 strand

(Calcd: 9829) (Calcd: 9829)


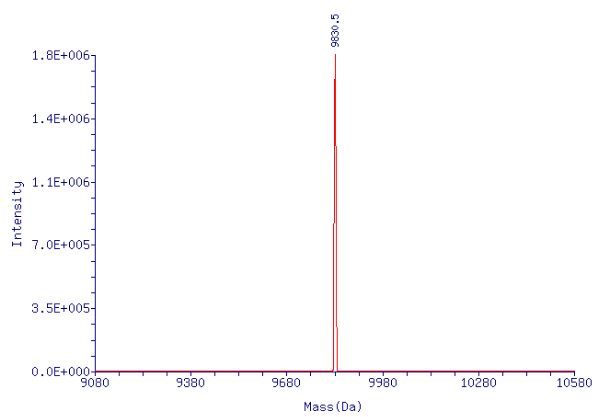

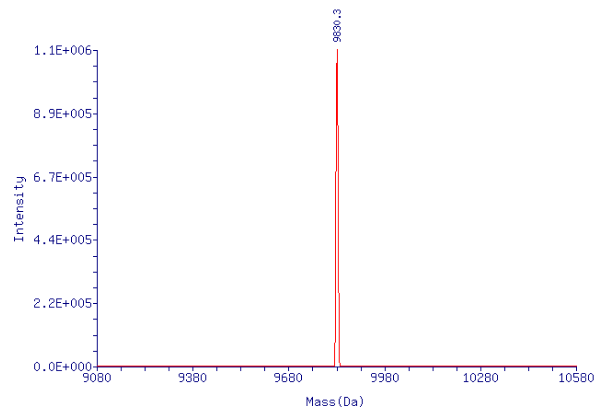


**Figure 18.** The ESI-MS of WW-15 strand **Figure 19.** The ESI-MS of WW-16 strand

(Calcd: 9829) (Calcd: 9829)


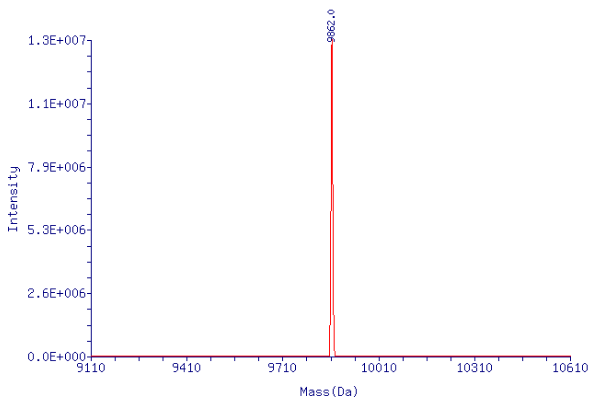

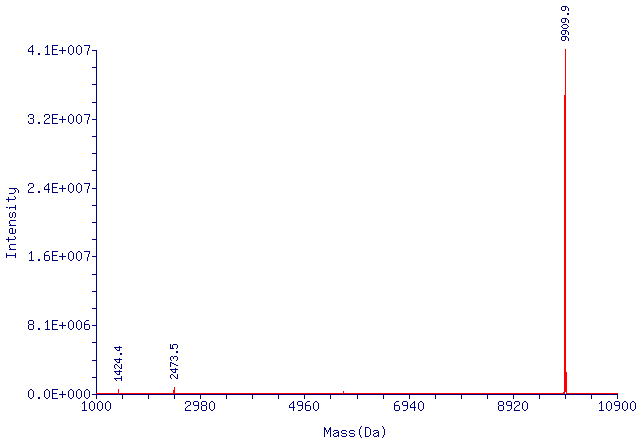


**Figure 20.** The ESI-MS of WW-18 strand **Figure 21.** The ESI-MS of WW-19 strand

(Calcd: 9861) (Calcd: 9909)


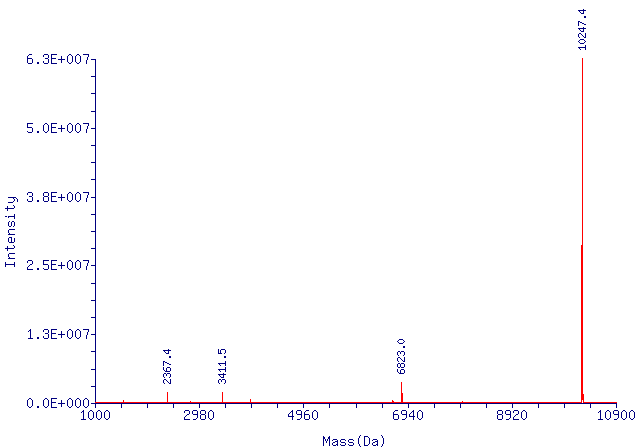

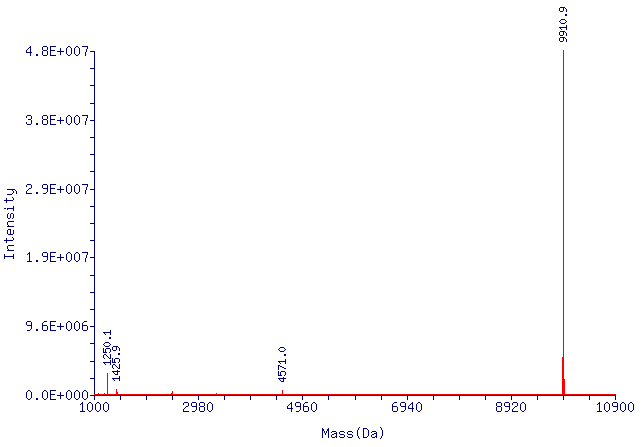


**Figure 22.** The ESI-MS of WW-20 strand **Figure 23.** The ESI-MS of WW-23 strand

(Calcd: 10246) (Calcd: 9909)


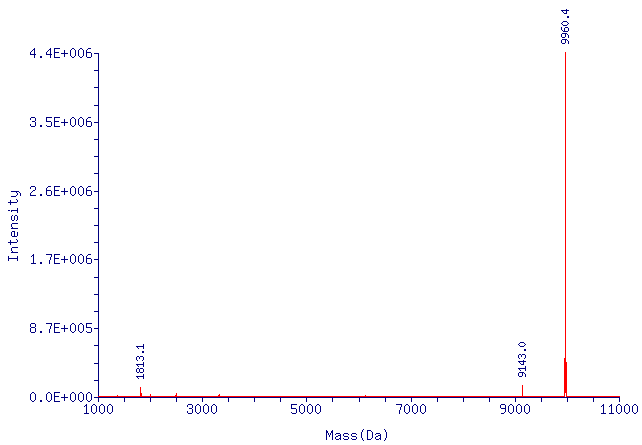

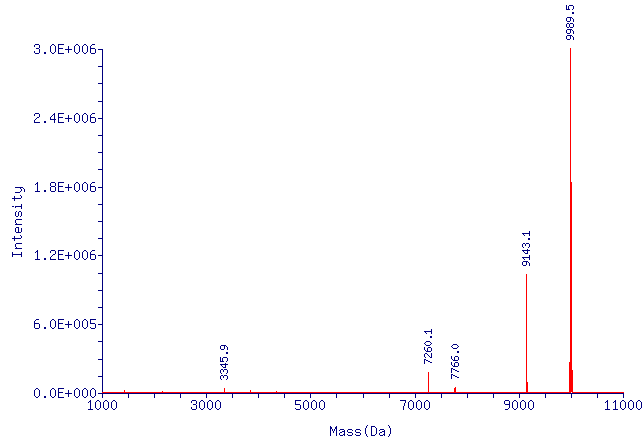


**Figure 24.** The ESI-MS of WW-24 strand **Figure 25.** The ESI-MS of 24-L1 strand

(Calcd: 9957) (Calcd: 9987)


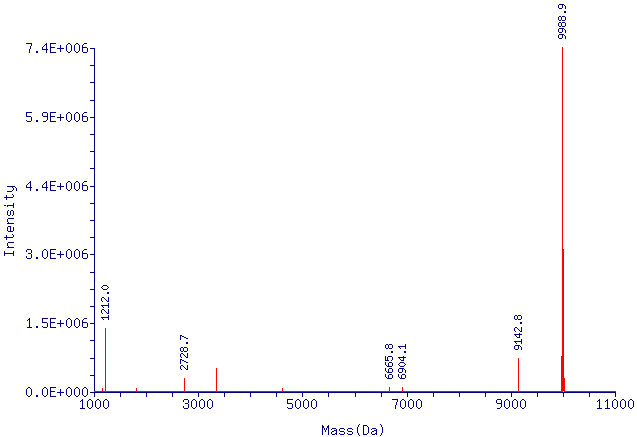

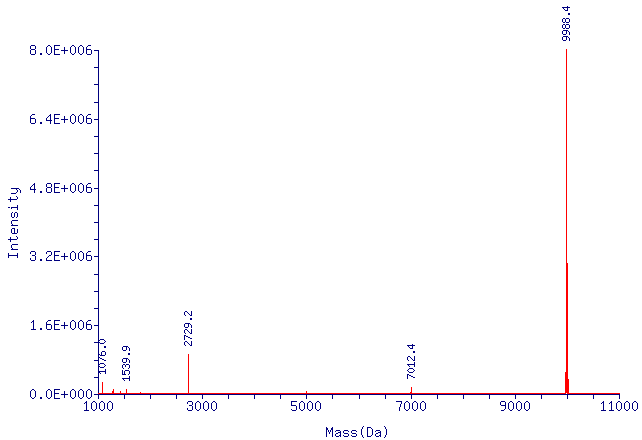


**Figure 26.** The ESI-MS of 24-L2 strand **Figure 27.** The ESI-MS of 24-L3 strand

(Calcd: 9987) (Calcd: 9987)


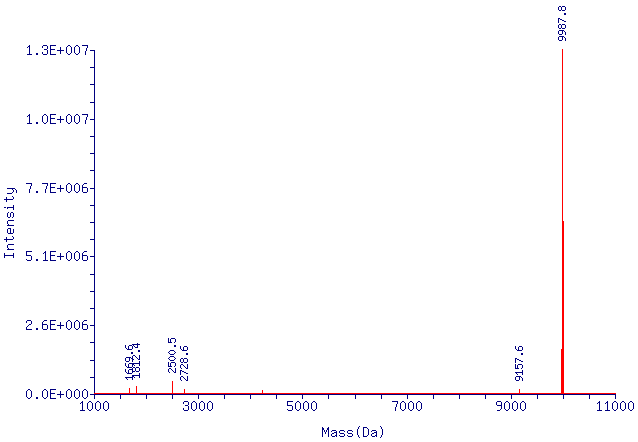

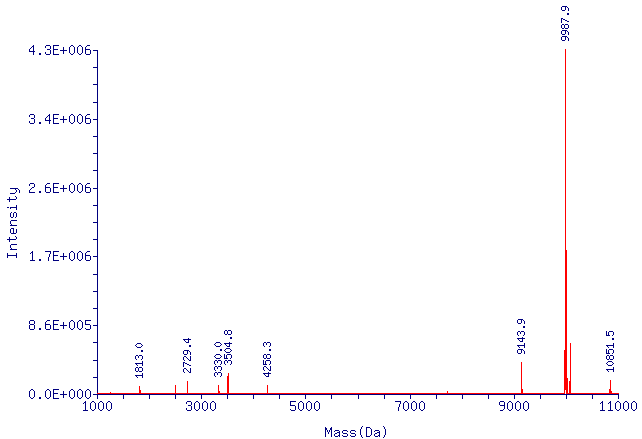


**Figure 28.** The ESI-MS of 24-L4 strand **Figure 29.** The ESI-MS of 24-L5 strand

(Calcd: 9987) (Calcd: 9987)


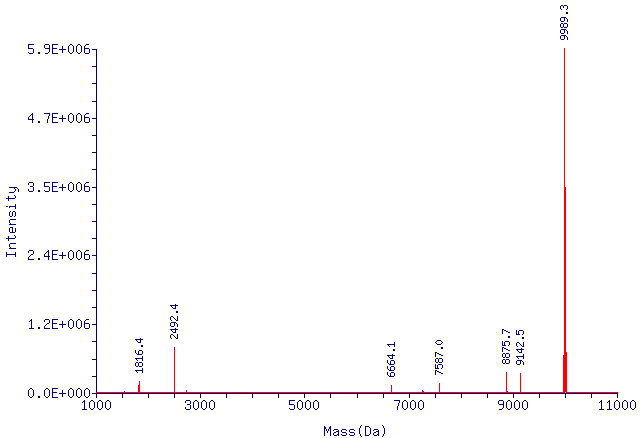

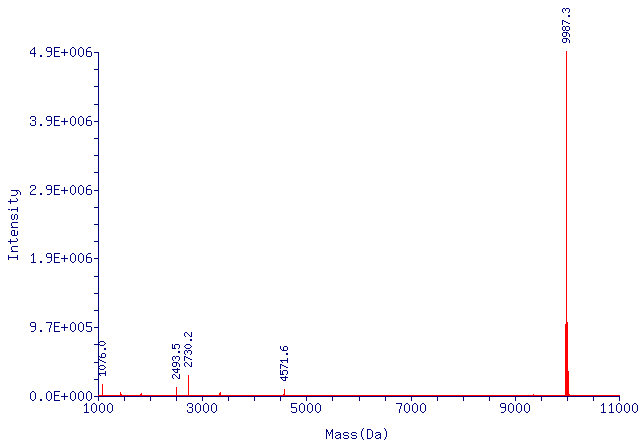


**Figure 30.** The ESI-MS of 24-L6 strand **Figure 31.** The ESI-MS of 24-L7 strand

(Calcd: 9987) (Calcd: 9987)


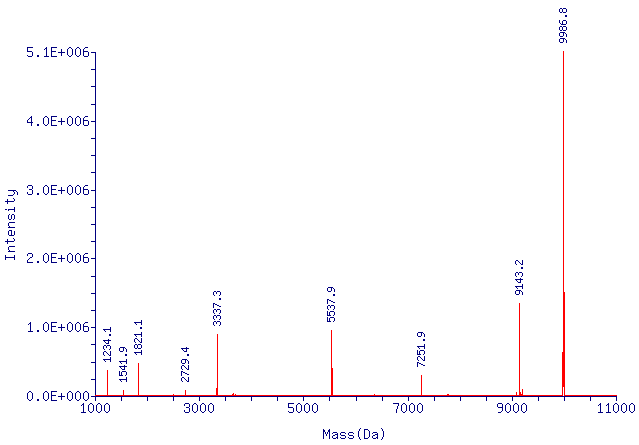

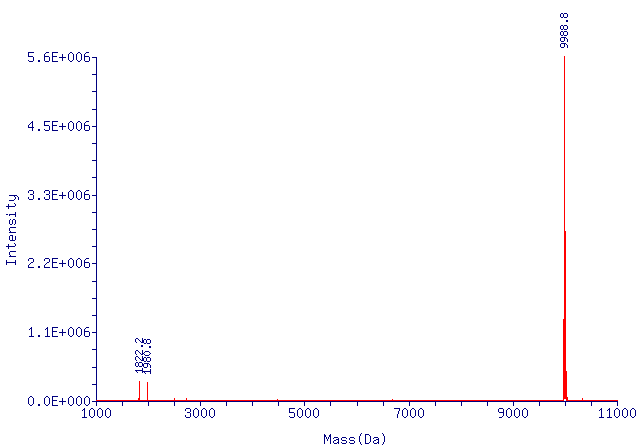


**Figure 312** The ESI-MS of 24-L8 strand **Figure 33.** The ESI-MS of 24-L9 strand

(Calcd: 9987) (Calcd: 9987)


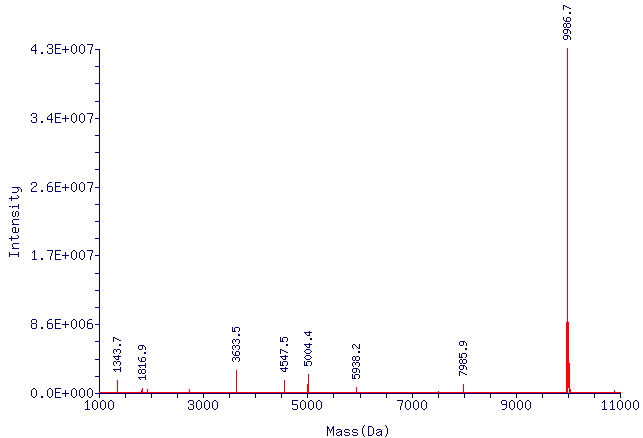

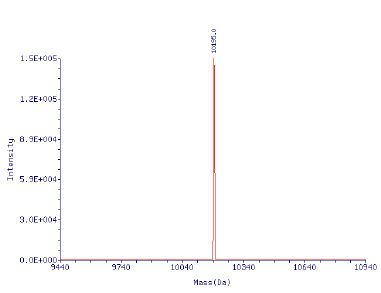


**Figure 34.** The ESI-MS of 24-L10 strand **Figure 35.** The ESI-MS of 24-M1 strand

(Calcd: 9987) (Calcd: 10194)


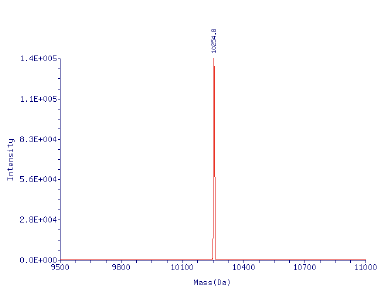

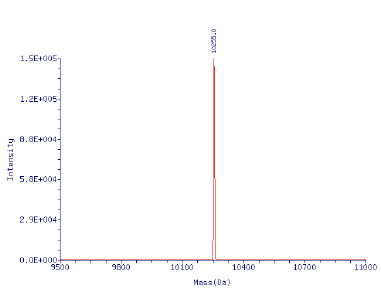


**Figure 36.** The ESI-MS of 24-M2 strand **Figure 37.** The ESI-MS of 24-M3 strand

(Calcd: 10254) (Calcd: 10254)


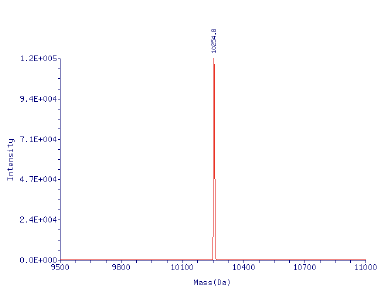

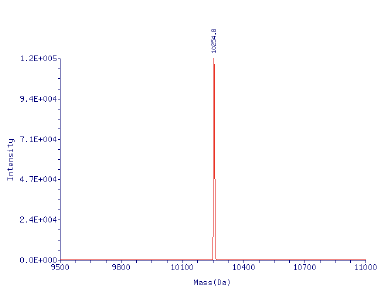


**Figure 38.** The ESI-MS of 24-M4 strand **Figure 39.** The ESI-MS of 24-M5 strand

(Calcd: 10254) (Calcd: 10254)


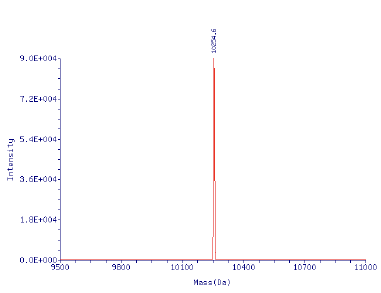

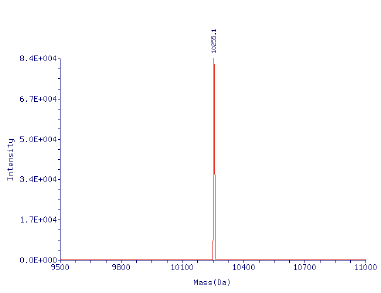


**Figure 40.** The ESI-MS of 24-M6 strand **Figure 41.** The ESI-MS of 24-M7 strand

(Calcd: 10254) (Calcd: 10254)


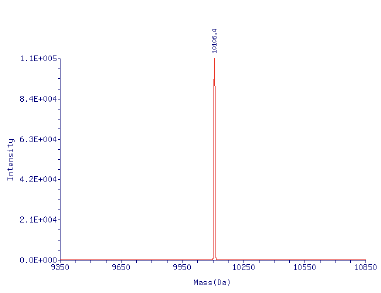


**Figure 42.** The ESI-MS of 24-M8 strand

(Calcd: 10106)


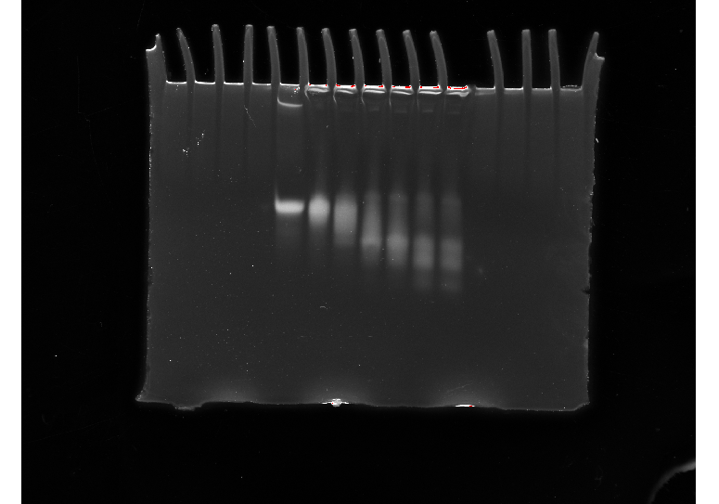

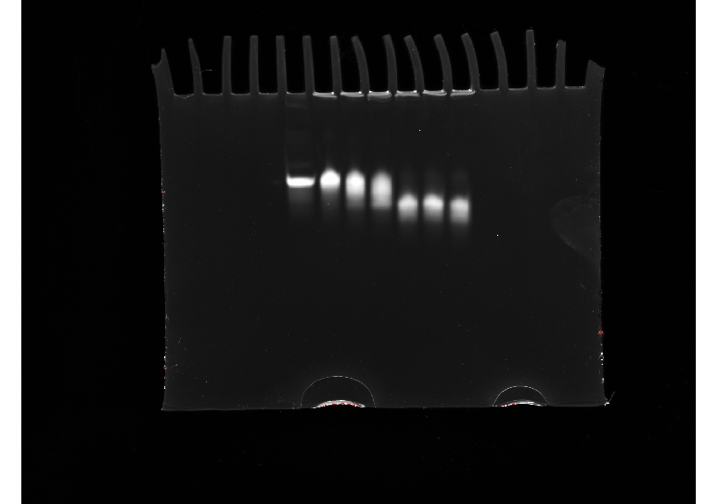


**Figure 43.** The gel pattern of WW-4 **Figure 44.** The gel pattern of BC15-31


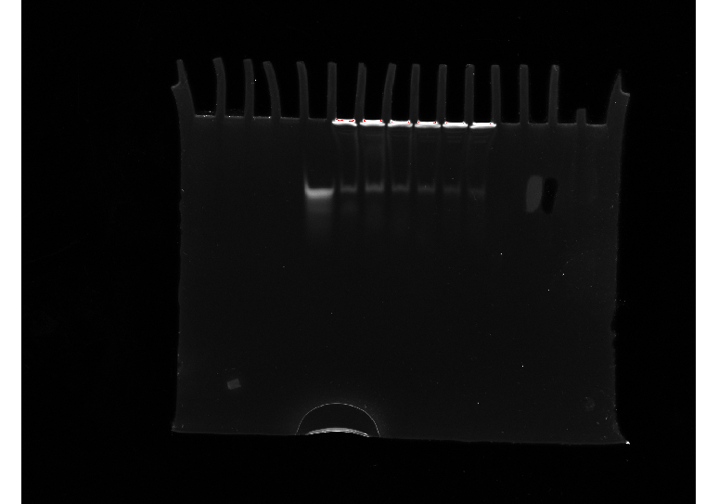

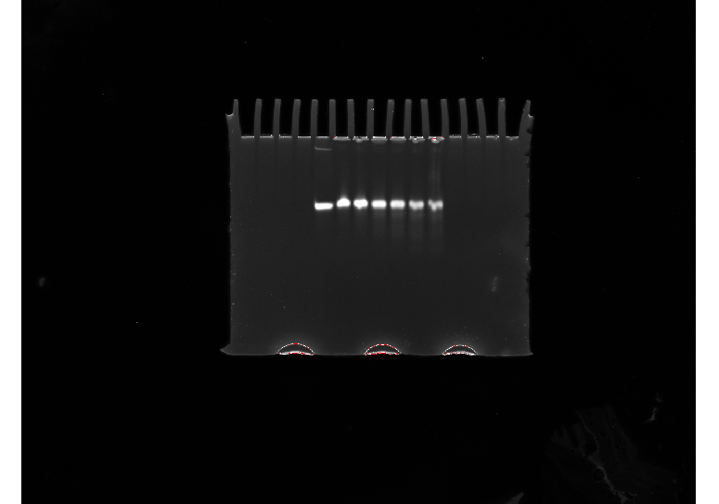


**Figure 45.** The gel pattern of WW-20 **Figure 46.** The gel pattern of WW-23


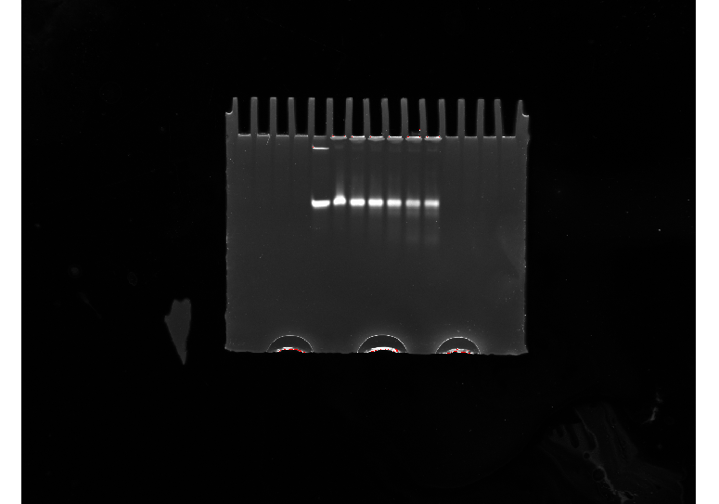

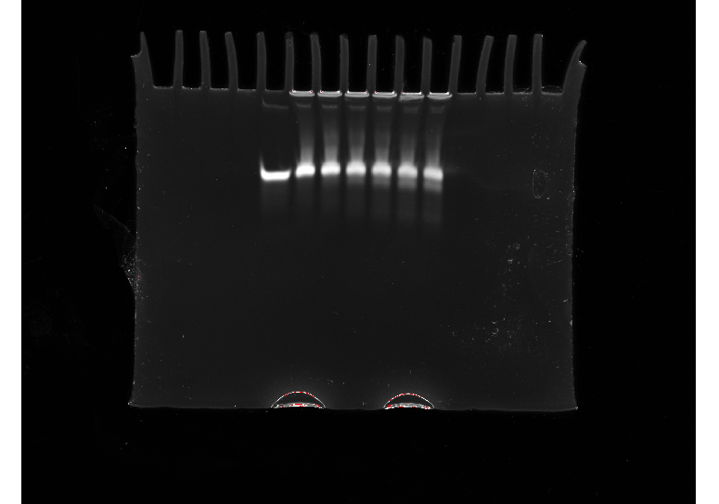


**Figure 47.** The gel pattern of WW-24 **Figure 48.** The gel pattern of 24-M6


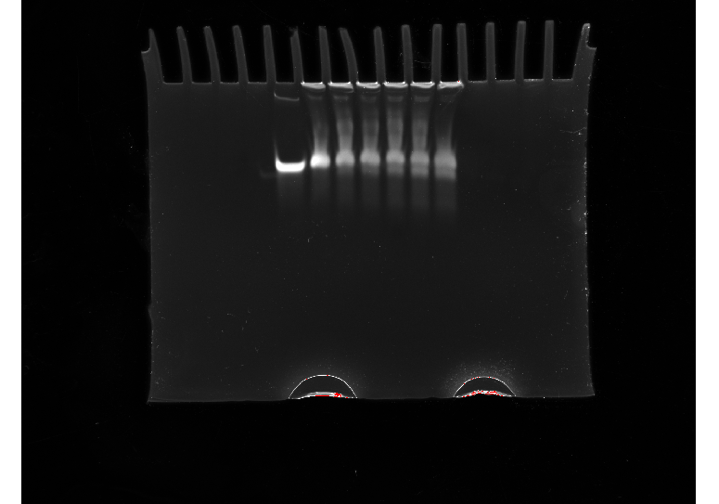


**Figure 49.** The gel pattern of 24-M8
